# Unveiling patterns: an exploration of machine learning techniques for unsupervised feature selection in single-cell data

**DOI:** 10.1093/bib/bbag006

**Published:** 2026-01-26

**Authors:** Nandini Chatterjee, Aleksandr Taraskin, Hridya Divakaran, Natalia Jaeger, Victor Enriquez, Catherine C Hedrick, Ahmad Alimadadi

**Affiliations:** La Jolla Institute for Immunology, 9420 Athena Cir, La Jolla, CA 92037, United States; Immunology Center of Georgia, Augusta University, 1410 Laney Walker Blvd, Augusta, GA 30912, United States; Immunology Center of Georgia, Augusta University, 1410 Laney Walker Blvd, Augusta, GA 30912, United States; Immunology Center of Georgia, Augusta University, 1410 Laney Walker Blvd, Augusta, GA 30912, United States; Immunology Center of Georgia, Augusta University, 1410 Laney Walker Blvd, Augusta, GA 30912, United States; Immunology Center of Georgia, Augusta University, 1410 Laney Walker Blvd, Augusta, GA 30912, United States; La Jolla Institute for Immunology, 9420 Athena Cir, La Jolla, CA 92037, United States; Immunology Center of Georgia, Augusta University, 1410 Laney Walker Blvd, Augusta, GA 30912, United States

**Keywords:** machine learning, unsupervised feature selection, single-cell data, pattern recognition, bioinformatics, artificial intelligence

## Abstract

The rapid evolution of single-cell technologies has generated vast, multimodal datasets encompassing genomic, transcriptomic, proteomic, and spatial information. However, high dimensionality, noise, and computational costs pose significant challenges, often introducing bias through traditional feature selection methods, such as highly variable gene selection. Unsupervised machine learning (ML) provides a solution by identifying informative features without predefined labels, thereby minimizing bias and capturing complex patterns. This paper reviews a diverse array of unsupervised ML techniques tailored for single-cell data. These approaches could enhance downstream analyses, such as clustering, dimensionality reduction, visualization, and data denoising, and reveal biologically relevant gene modules. Despite their advantages, challenges such as data sparsity, parameter tuning, and scalability persist. Future directions include integrating multiomic data, incorporating domain-specific knowledge, and developing scalable and interpretable algorithms. By addressing these challenges, unsupervised ML-based feature selection promises to revolutionize single-cell data analysis, driving unbiased insights into cellular heterogeneity and advancing biological discovery.

## Introduction

Recent advances in cellular biology have revolutionized the field. New technologies such as sequencing, mass spectrometry, imaging, flow cytometry, and mass cytometry now enable detailed, multimodal analysis of individual cells. These methods provide extensive data on the genome, transcriptome, proteome, methylome, chromatin, histone modifications, and spatial information. A major challenge is the large volume of cellular-level data, which incurs high computational costs and makes it impractical to include all features. Many features lack informative value and may add noise, compromising analysis [1]. Therefore, selecting informative features is essential for dimensionality reduction, clustering, visualization, and biological comparisons [2, 3]. The common strategy is to use highly variable genes (HVGs), assumed to carry more information. However, while variability highlights differences between cells, it can obscure within-cell patterns, especially interactions among features.

This challenge extends to biological interpretation, as insights from cell types, differentially expressed genes, and pathways depend on comparisons across factors such as disease status, sex, and tissue type. These factors can bias results; for instance, cell type 1 versus type 2 may differ from cell type 1 versus type 3. Managing hundreds of such comparisons across diverse data types, cell types, and metadata is highly complex.

While supervised feature selection methods can offer high accuracy when the target labels are well defined, they can also introduce bias or limit discovery in single-cell settings. In contrast, unsupervised feature selection evaluates features in the absence of prior labels or predefined groupings [4]. These methods, which analyze data without prior knowledge of the cell [5], can detect distinct patterns in individual cells and provide several distinct advantages [6]. Unsupervised methods can uncover latent structure in the data, such as subtle gradients of cell states, rare or transitional populations, or coexpression modules, that may be masked when forcing genes to align with existing annotations. In addition, because they do not depend on supervised labels, these methods are more flexible and generalizable across datasets whose labels differ in quality or completeness. They can detect informative features independently of how cells have been annotated or how metadata have been structured. Furthermore, by emphasizing multivariate relationships, manifold or latent factor structure, or the preservation of local or global geometry, unsupervised selection can reduce redundancy and noise more effectively than simple variance-based filtering, leading to cleaner embeddings, better clustering resolution, and more interpretable genes [7].

**Figure 1:**
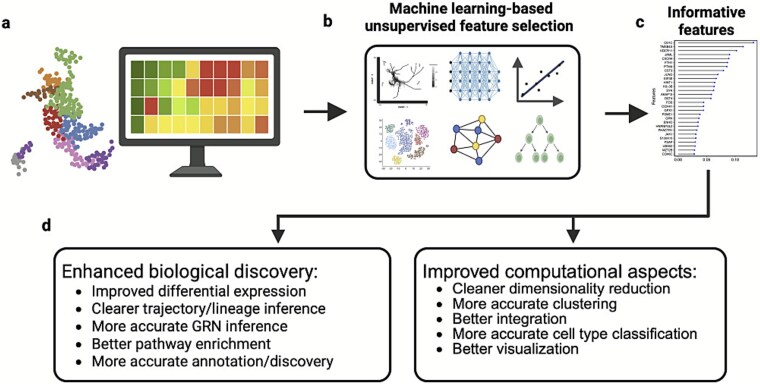
Feature selection using ML improves single-cell analysis. (a) High-dimensional single-cell data. (b) Unsupervised ML techniques are applied to identify informative features. (c) Top informative features are selected for downstream analysis. (d) Examples of computational and biological aspects that can be improved using an informative gene list.

Given the limitations of supervised methods, recent studies increasingly emphasize analyzing each cell’s profile directly to reduce bias from external factors. Figure 1 illustrates the general concept of feature selection from high-dimensional data to improved computational/biological outcomes. The analysis starts with the initial single-cell dataset (Fig. 1a), followed by the application of unsupervised machine learning (ML)-based feature selection to identify intrinsic data structure without predefined labels (Fig. 1b). This process yields a set of informative features (Fig. 1c), leading to improved biological insight and computational performance in downstream analyses (Fig. 1d). Once identified, these patterns can then be compared across cells or metadata groups, ensuring feature discovery is independent of confounding factors.

Our literature review shows that comprehensive resources on this topic are scarce. This article introduces unsupervised ML methods tailored for identifying informative cellular features, efficiently pinpointing variables with high informational value to support both computational and biological interpretation. Specifically, this review addresses how unsupervised feature selection can be used to select informative features to reveal biologically meaningful patterns, improve computational efficiency, and enhance interpretability in single-cell data. Building on this foundation, we classify and describe these methods according to their underlying computational principles and typical applications, emphasizing how they capture complex, multivariate patterns beyond traditional variance-based strategies. Rather than providing a comprehensive survey of software implementations, our focus is on the conceptual understanding and methodological frameworks that underpin unsupervised feature selection. As analytical packages and tools continue to evolve, these core principles will remain relevant and broadly applicable across future developments in the field. Our work aims to serve as a reference and catalyst for advancing single-cell analysis by providing a structured overview of modern methods and their applications.

## Unsupervised machine learning techniques for feature selection

### Linear dimensionality reduction methods

#### Principal component analysis

Principal component analysis (PCA) is a widely employed technique in multivariate analysis for examining datasets and gaining initial insights into their underlying structure. When dealing with multiple observations and variables for each object, PCA serves various purposes, such as streamlining data, diminishing dimensionality, identifying outliers, and selecting relevant variables [8]. PCA is employed in single-cell data analyses to reduce the dimensionality of datasets, capture the most significant sources of variation, and simplify downstream analyses such as clustering and visualization [9, 10]. In unbiased, unsupervised analysis, PCA’s adaptability, without predefined functions or distribution assumptions, aligns with revealing hidden patterns and structures in a label-free manner [11]. Despite its capabilities, it is important to note that PCA does not capture nonlinear relationships [12].

The principle involves standardizing data, computing the covariance matrix, and deriving eigenvalues and eigenvectors, or performing singular value decomposition. Once principal components are obtained, scores (cell coordinates) and loadings (variable contributions) are calculated [11]. PCA loadings show each variable’s influence on components, with higher absolute values indicating a stronger impact. Ranking variables by loading magnitude identifies the most informative features for further computational and biological analyses.

While PCA loadings are widely used for feature selection, interpreting PCs in gene expression data is difficult due to complex gene combinations. Sparse PCA improves interpretability by focusing on fewer genes [13]. Loadings can be assessed using methods such as cutoff rules, broken-stick, correlation thresholds, resampling, randomization, bootstrapped broken-stick, and parallel analysis [14]. Multiple implementations of PCA exist and are tailored to different data characteristics and objectives.

#### Independent component analysis

Independent component analysis (ICA) is a powerful unsupervised learning technique that can be applied to single-cell RNA sequencing (scRNA-seq) data for feature extraction and selection. ICA decomposes the gene expression matrix into statistically independent components (ICs), each capturing a distinct underlying signal potentially linked to biological variability. Unlike methods that assume specific data distributions, ICA is nonparametric and identifies components based on statistical independence, making it well suited for complex, heterogeneous single-cell datasets. Each IC is characterized by gene loadings that reflect how much each gene contributes to the component. Genes with the highest absolute loadings across ICs are likely involved in driving key biological processes, such as specific cell states, signaling pathways, or transcriptional programs. By ranking genes on the basis of their contributions to multiple ICs, ICA facilitates the selection of highly informative genes for downstream analyses such as clustering, classification, or pathway enrichment [15, 16].

While traditional ICA assumes linear mixing and non-Gaussian sources, recent adaptations have extended its applicability to scRNA-seq by addressing challenges such as high dimensionality, convergence issues, and the need for prior dimensionality reduction, often via PCA. These improvements have enhanced the utility of ICA for identifying biologically meaningful features, improving single-cell clustering, and integrating data across batches [17–19].

#### Sparse principal component analysis

Sparse principal component analysis (Sparse PCA) addresses the challenges in traditional PCA, including the difficulty in interpreting the component scores, nonuniqueness, and instability of the component loadings/weights [20]. It aims to identify a sparse set of features that captures the most important variations in high-dimensional biological data. This method reduces dimensionality and extracts meaningful patterns in datasets with many variables. Sparse PCA works on the idea that the principal components, which are linear combinations of the original variables, can be represented by a small number of key features. In contrast, the remaining numbers are set to zero. The sparse PCA algorithm first computes the covariance matrix or correlation matrix. Then, an optimization problem is formulated to find the principal components that maximize the variance while enforcing sparsity in the loadings [21]. This can be achieved through techniques such as L1 regularization or thresholding.

Sparse PCA enhances interpretability and scalability by selecting key variables from high-dimensional data while reducing computational complexity. Its sparsity-inducing penalty improves efficiency, making it well suited for biological datasets [22]. By ranking variables based on their absolute loadings, sparse PCA identifies key features while reducing dimensionality and improving interpretability, making it particularly useful for high-dimensional biological data. This algorithm may face challenges in selecting optimal regularization parameters, which often require cross-validation. It may also struggle when data lacks sparsity or when relevant variables are not well distinguished from irrelevant variables.

#### Consensus principal component analysis

Consensus PCA (CPCA), introduced by Wold *et al.* [23] and later refined [24], extends PCA to handle multiple data blocks. It creates a ‘super score’ vector that integrates information across blocks, revealing patterns hidden in isolated analyses. CPCA outputs include block loadings (variable importance within each block), block scores (object projections within blocks), and weights (each block’s influence on the superposition score, emphasizing significant contributions to the overall representation) [23, 24].

In practice, CPCA can also be used as a stability-based approach, in which PCA is repeated across subsampled or perturbed datasets, and the results are aggregated to identify features with consistently high loadings. After performing consensus PCA, the results are aggregated, for example, by averaging the absolute values of loadings or assessing the consistency of feature loadings across subsampled runs. Features are ranked by average importance or consistency across subsets, with top features showing greater stability and contributions. Consensus PCA enhances feature selection by improving robustness, preventing overfitting, ensuring stability with consistently selected features, and reducing noise by minimizing the impact of uninformative features, making it valuable for sparse, noisy single-cell data analysis. It’s worth mentioning that consensus PCA is more complex and time-consuming than regular PCA, as it may involve subsampling and aggregating results.

#### Application to single-cell data

In single-cell analysis, linear dimensionality reduction methods help highlight the genes that truly drive differences between cells. PCA loadings point to genes underlying major transcriptional shifts, often linked to cell identity, activation, or cell cycle. ICA goes a step further by separating independent biological programs, making it easier to find gene sets related to specific pathways or states that variance alone might miss. Sparse PCA makes these patterns more interpretable by selecting only the most informative genes instead of spreading the signal across thousands. Additionally, consensus PCA stabilizes this process by identifying features consistently important across subsampling or perturbations. Together, these approaches yield clearer, more focused gene sets that improve downstream clustering and biological interpretation.

### Nonlinear dimensionality reduction methods

#### Uniform manifold approximation and projection

Uniform manifold approximation and projection (UMAP) is a dimensionality reduction and visualization method that preserves global structure and enables fast processing [25, 26]. It constructs a high-dimensional weighted graph and optimizes a low-dimensional layout via a force-directed algorithm, using insights from algebraic topology and Riemannian geometry. Connectivity is determined locally using k-nearest neighbors, avoiding isolated points and mitigating the ‘curse of dimensionality.’ Key parameters include the number of neighbors, which balances local versus global structure, and the minimum distance, which controls embedding tightness versus broader topology [25].

Although UMAP is primarily used for visualization, it can be repurposed for unsupervised feature selection by quantifying the strength of each gene’s contribution to UMAP embedding. This can be done by correlating gene expression profiles with UMAP coordinates or using regression to predict these coordinates from gene expression, treating the resulting coefficients as feature importance scores. To aggregate importance across multiple UMAP dimensions, genes can be ranked based on the sum or average of absolute correlations or regression weights. Alternatively, genes consistently ranked in the top N features across dimensions can be prioritized. These strategies enable the identification of genes that drive meaningful biological variation without relying on a label [27, 28].

#### Topological data analysis

The main goal of topological data analysis (TDA) is to leverage a dataset’s topological and geometric structure. This can be understood by considering a dataset as a cloud of points embedded in an n-dimensional geometric space. Rather than exploring the data directly, TDA examines the shape of this point cloud to characterize our dataset [29]. In TDA, persistent homology (PH) captures qualitative features of a dataset by analyzing how topological structures such as connected components, loops, and voids persist across scales [30, 31]. In single-cell data, cells are represented as a point cloud with genes as dimensions, and PH identifies genes that strongly influence topological features. These genes, reflecting key biological patterns, can then be selected for downstream analyses.

Chordal graphs, such as triangulated maximally filtered graphs, select relevant features based on network connectivity in topologically constrained graphs [32]. Alternatively, PH with sliding window embeddings can capture patterns in vector-valued time series [33], and gradient descent is used to optimize feature weights, prioritizing those that most influence topological structures like connected components or loops.

Mapper, a TDA algorithm, integrates dimensionality reduction, clustering, and graph techniques to generate a lower-dimensional representation of complex datasets [34, 35]. The algorithm first applies a filter function (lens) to project data into a lower-dimensional space, then divides this space into overlapping bins (covers). Each bin is independently clustered using the original high-dimensional data, grouping similar points into clusters (nodes). Finally, the clusters (nodes) that share at least ‘i’ data points (user-defined threshold) will be connected, thus generating a mapper graph. This graph can be used for unsupervised feature selection: nodes reveal genes with high variance or differential expression that define cell states, edges highlight genes driving transitions between clusters, and centrality metrics such as betweenness identify genes with key structural roles in the graph, providing insights into both cellular identity and dynamics.

Unsupervised feature selection with TDA and Mapper can identify key genes driving cell states and transitions in scRNA-seq data without labels. However, it faces challenges, including sensitivity to parameters (e.g. lens function, cover size), difficulty interpreting topological features, and robustness issues due to noise and sparsity inherent in single-cell data.

#### Locally linear embedding

Locally linear embedding (LLE) is an unsupervised, manifold feature selection algorithm that aims to preserve the local structure of high-dimensional data in a lower-dimensional space [36]. It operates on the premise that the data’s local structure is effectively captured by linear relationships between neighboring data points. By maintaining these local relationships, LLE can uncover the intrinsic low-dimensional structure of the data [37].

LLE identifies each point’s nearest neighbors and computes weights to reconstruct points by minimizing reconstruction error, which is the difference between the original data point and its linear combination of neighbors. A low-dimensional embedding is then obtained by optimizing point positions [37]. By leveraging local relationships, LLE extracts meaningful features, handles nonlinear manifolds, scales well to large datasets, and provides a low-dimensional representation that facilitates further analysis.

LLE-based methods, such as graph-preserving feature selection embedding LLE, the LLE score, and graph regularized local linear embedding, have been widely applied in unsupervised feature selection. These approaches select features based on their ability to preserve local data structures, minimize deviations from the original topology, or learn feature selection matrices that maintain neighborhood relationships [38].

The result of LLE depends on defining optimal parameters, such as the number of neighbors and the dimensionality of the embedding space [38]. It may not perform well in scenarios where the local structure of the data is poorly defined or when outliers or noise are present.

#### Isometric mapping

Isometric mapping (Isomap), introduced in 2000, unlike many other clustering techniques that rely on Euclidean distances, is an efficient nonlinear dimensionality reduction method that preserves the geometric structure of the data [39]. Isomap considers geodesic distances instead of straight-line distances in high-dimensional space. This technique assumes that the underlying data lies on a low-dimensional manifold embedded within the high-dimensional space. To achieve this, Isomap constructs a neighborhood graph by connecting each data point to its nearest neighbors. The graph is then utilized to calculate geodesic distances, which represent the shortest paths between two points along the manifold. Isomap subsequently utilizes these geodesic distances to embed high-dimensional data points into a lower-dimensional space [39, 40].

One approach to identify the most informative genes is to calculate gene scores by assessing the correlation between each gene’s expression and the low-dimensional Isomap coordinates. Alternatively, feature importance can be determined via a regression model trained to predict Isomap components from gene expression, allowing for the selection of genes that contribute most to the manifold structure.

Isomap is robust to noise and outliers in the data, can be used as a dimensionality reduction technique, enhances clustering performance, can improve and be applied to the computational biology field, and is user-friendly in terms of having only a few parameters to choose from. Challenges include selecting the number of neighbors, high computational cost for large datasets (mitigated by prior feature selection), and its nondeterministic nature, which can produce different embeddings for the same data [40–43].

#### Diffusion maps

Diffusion maps are a spectral method for nonlinear dimension reduction adapted for scRNA-seq analysis, particularly for assessing cell differentiation trajectories [44]. This algorithm offers three key advantages over classical dimensionality reduction methods such as PCA: it is nonlinear, making it better suited for data that do not lie on linear manifolds; it is robust against noise, as the diffusion process smooths out small variations; and it preserves both local and global structures, which may be represented in branches [44–46]. This technique leverages a distance metric to determine relationships between data points, in this case, cells, based on their gene expression profiles. Initially, it constructs a weighted graph from pairwise similarities between cells on the basis of a kernel function, such as a Gaussian kernel. Then, a Markov transition matrix is derived from the similarity graph, which represents the probabilities of moving from one point to another in a diffusion process. Eigenvalue decomposition transforms this matrix into new coordinates, where the top eigenvectors capture the dominant diffusion patterns. Keeping only the leading eigenvectors yields a low-dimensional embedding that preserves the most important diffusion-driven relationships between cells [44–46].

Diffusion maps do not provide direct gene loadings, such as PCA, but allow for the identification of gene contributions through postprocessing. Genes can be assessed by correlating their expression with the leading diffusion components or by constructing a gene-based diffusion map via a gene–gene similarity matrix. Feature importance can also be evaluated through regression models to predict diffusion components from gene expression. These methods help identify key genes that drive the diffusion structure and are likely key markers of biological variation. Despite their strengths, diffusion maps can be challenging when the appropriate parameters are optimized, such as when an appropriate kernel width is selected. Diffusion maps have been implemented for single-cell data analysis via various tools, such as the destiny package [47].

#### Application to single-cell data

Nonlinear dimensionality reduction methods help us identify important genes in single-cell data by capturing patterns that linear methods can’t. Techniques like UMAP can point to genes that shape how cells cluster or spread out in the embedding, either locally or across the full landscape. TDA methods, such as PH and Mapper, highlight genes associated with branches, loops, or rare cell states, patterns that often reflect real biological transitions. Methods such as LLE and Isomap highlight genes that help preserve the underlying data structure, especially when cells follow smooth developmental or spatial paths. Diffusion maps are particularly helpful for identifying genes associated with gradual changes or lineage progression. Taken together, these nonlinear approaches help identify gene sets that reflect the true complexity of cellular behavior.

### Representation learning and latent factor models

#### Nonnegative matrix factorization

Nonnegative matrix factorization (NMF) is a ML technique for image analysis, speech recognition, language processing, and dimensionality reduction. The nonnegative constraint in the factorization of the data matrix enhances interpretability. In addition to dimensionality reduction, NMF enables feature extraction. It assumes that each document is a linear combination of topics and that each topic is a linear combination of terms or features. In other words, extracted topics in NMF refer to the nonnegative components or patterns identified in the data through the factorization process of the original attribute set, and these components could have applications in various domains where nonnegativity and interpretability are important [48]. The factorization is usually found with an Expectation–Maximization algorithm or stochastic gradient descent.

Based on the analysis of the factorized matrices, we can select the original features with the highest coefficients. These selected features are expected to carry the most information and be the most discriminative for downstream tasks such as clustering, classification, or regression. After identifying important features in each component, we can either select shared features across patterns or those uniquely associated with each topic. In single-cell data, shared features likely represent core biological processes or genes consistently expressed across cell states, while unique features may capture cell-type-specific markers, regulatory programs, or transient states that distinguish different cellular populations. Commonly applied in cancer genomics, NMF identifies mutational signatures and gene programs [49].

Because it is suitable for smaller datasets, NMF capitalizes on vector nonnegativity. Unlike PCA, its coefficient matrix provides approximations, and minimizing parameters is NP-hard but solvable numerically. Since the results are not deterministic, it is possible to obtain different results each time the model is run, even on the same dataset; however, attempts have been made to improve these results [48]. Nevertheless, NMF efficiently learns topics through direct decomposition of the document-term matrix, facilitating statistical analysis of multivariate data [50].

#### Topic modeling

Topic modeling was originally used in text analysis to identify and extract latent topics by analyzing the co-occurrence patterns of words across the entire document collection without the need for predefined tags or training data [51]. Topics assigned automatically to the documents can be used to extract meaningful features without the need for manual annotation or supervision.

Latent Dirichlet Allocation (LDA) is the most common algorithm for topic modeling. It estimates two sets of variables: document-topic proportions and topic-word distributions. Initially, the algorithm assigns random topic proportions to each document and random word distributions to each topic. It then iteratively updates these variables based on the observed word occurrences in the documents. The outcome is a set of topics, each represented by a distribution of words, and each document is assigned a set of topics. This inherently reduces the enormous dimension of the original biological data space into a smaller latent topic space. Although the topics themselves could be treated as dimensionality reduction in downstream steps, we can also use the resulting model to select the most informative features [52]. We can identify informative features by analyzing the likelihood of words appearing in specific topics or a set of topics. Features with a higher probability in multiple topics can be considered conserved features.

In contrast, the topics that have different probabilities in distinct topics can be identified as discriminative features [53]. Topic modeling can handle high-dimensional, sparse data efficiently in a reasonable amount of time. Thus, it can be used across a wide range of data types, including gene expression profiles, protein–protein interaction networks, clinical datasets, or image analyses such as magnetic resonance imaging (MRI) [54]. One example of topic modeling applications is scATAC-seq data, where LDA-based approaches, such as the SnapATAC pipeline [55], model cells as ‘documents’ and peaks as ‘words.’ Additionally, term frequency-inverse document frequency (TF-IDF) transformation followed by latent semantic indexing [56] is commonly used to highlight cell type-specific accessible regions and reduce noise, facilitating effective feature selection in single-cell chromatin data. However, the results of topic modeling are sensitive to the choice of hyperparameters, such as the number of topics, which is still less explored using optimization on biological data, thus indicating a long and challenging path ahead.

#### Dictionary learning

Dictionary learning, or sparse coding, represents data as a linear combination of repetitive elements called a dictionary [57]. It assumes that a few dictionary elements can sparsely represent data. One of the method’s main strengths is that the fewer dictionary elements needed to represent the data, the more effective and meaningful the resulting dictionary tends to be.

Dictionary learning effectively identifies the most informative features within a dataset by iteratively optimizing a dictionary of elements and sparse representation coefficients via regularization techniques that promote the selection of relevant features [58]. By learning from the data adaptively, the algorithm can discern meaningful patterns and structures, extracting key features that best represent the underlying information. One of its notable strengths is its adaptability to different data types, making it suitable for real-world applications. It excels in capturing the underlying structure of high-dimensional data and handling noisy or incomplete data [59, 60]. However, traditional dictionary learning algorithms may face challenges such as the limited interpretability of learned features, sensitivity to initialization, and computational complexity, particularly for extensive datasets or highly nonlinear data distributions. In scenarios where data lacks inherent structure or has high dimensionality, dictionary learning may struggle to extract meaningful features accurately. Despite these challenges, dictionary learning has been successfully applied in computational biology. For example, it has been employed for dimension reduction, pseudotime estimation [61], and the integration of multiomic datasets via tools such as Seurat v5 in single-cell RNA sequencing data analysis [62]. However, researchers must consider these limitations and challenges when applying dictionary learning to real-world biological datasets.

#### Application to single-cell data

Features identified through NMF, topic modeling, and dictionary learning can be applied to single-cell analysis to reveal biological structure. High-weight genes from NMF indicate gene programs that define cell states, aiding in clustering, marker identification, and tracking shared programs. Topic modeling highlights genes or regions within latent topics, useful for annotating regulatory modules and identifying markers. Dictionary learning isolates sparse features that summarize variation, supporting pseudotime ordering, and multiomic integration. These strategies reduce complex data into interpretable gene sets or elements, enhancing analysis robustness and resolution.

### Regularization-based methods

#### Least absolute shrinkage and selection operator and elastic net

Least absolute shrinkage and selection operator (LASSO), elastic net (ENET), and their variants are regularization techniques used in ML modeling. LASSO regression, which is favored in domains such as genomics with massive datasets, uses the ℓ1 penalized least squares criterion and tends to produce sparse solutions, effectively setting some coefficients to zero [63]. It naturally performs feature selection while keeping the genes with nonzero coefficients. It can be less robust when predictors are highly correlated, arbitrarily choosing one and ignoring others. The ENET extends the lasso designed for robustness to strong predictor correlations, which is particularly useful in high-dimensional data analysis. It employs a mixture of ℓ1 (lasso) and ℓ2 (ridge regression) penalties for stability [64]. It allows for some level of sparsity while accommodating groups of correlated genes.

Typically, applied in supervised learning, these methods aim to predict a target variable. When used for unsupervised feature selection, all features can be treated as responses for prediction. LASSO and ENET regularization encourage some coefficients to be zero, selecting a subset of features to identify relevant patterns or structures within the data without an external target variable guiding the process. The next step is to count the occurrences of each selected feature, rank them based on frequency, and construct our final list of informative features.

Algorithm choice depends on variable relationships, desired feature number, and variable-to-sample ratio. ENET works well when features outnumber observations, whereas LASSO performs poorly in such cases [64]. Penalized regression is computationally efficient but may require preliminary filtering for large datasets [65].

#### Graph Laplacian regularization

Graph Laplacian regularization (GLR), which is commonly used in graph-based models (e.g. graph neural network) to prevent overfitting, can address unsupervised feature selection by preserving the local structure of a dataset through a similarity graph [66, 67]. GLR employs a graph Laplacian matrix as a regularization term during the optimization process, where the goal is to identify features that both minimize the loss function and preserve the data structure.

One of the main challenges of applying GLR to feature selection is its sensitivity to noise. Tang *et al.* [68] address this issue by proposing an approach that adopts L1-norm-based regularization to promote sparsity and thus reduce noise impact. To apply GLR for feature selection in single-cell data, we first construct a similarity graph, where nodes represent cells and edges reflect gene expression similarity. GLR is then used as a regularization term to preserve the local structure of the data while enforcing sparsity to filter out noisy or irrelevant genes. When optimized via gradient descent or proximal methods, the objective function balances feature relevance, structure preservation, and sparsity. Gene weights are derived from the sparse solution of an optimization problem that incorporates GLR, which ranks genes by their contribution to preserving the data’s local structure. The top-ranked genes are selected as the most informative [66–68].

GLR in feature selection is valuable for three key reasons: it preserves the local geometric structure of data, reduces redundancy, and improves robustness to noise by promoting sparsity and filtering out irrelevant features. This makes GLR particularly useful for identifying patterns in complex datasets and ensuring more accurate and interpretable models, whether during feature selection or prediction with graph-based models.

#### Application to single-cell data

Features selected with LASSO, ENET, or GLR can make single-cell analyses cleaner and more focused. Genes that retain nonzero coefficients in LASSO/ENET tend to capture the strongest or most informative signals, helping remove redundancy and reduce noise before clustering or visualization. GLR-selected genes, on the other hand, preserve the structure of the cell–cell similarity graph, which is especially helpful for neighborhood-based methods like UMAP, trajectories, and graph clustering. Using these smaller, more meaningful gene sets often makes it easier to separate cell types, detect subtle transitions, and build more stable downstream models. Overall, these methods help highlight the genes that truly matter while improving interpretability and consistency across analyses.

### Tree-based and ensemble feature selection methods

#### Random forest

The random forest (RF) algorithm stands out as one of the most widely used classification models, featuring a collection of decision tree classifiers. In this ensemble, each tree provides a singular vote to establish the predominant class for a given input [69]. This potent classifier utilizes bagging and random feature selection to achieve quick and effective results. The algorithm ensures clarity by internally prioritizing crucial features during tree construction, minimizing the impact of irrelevant features [70].

While RF is utilized primarily as a supervised ML model, attempts have been made to employ it in an unsupervised manner, particularly for clustering unlabeled data [71, 72]. To execute a RF via a semisupervised approach, the original data are considered class 1, and a synthetic second class is created by sampling from the univariate distributions of the original data, thereby destroying its dependency structure. This enables the application of RF algorithms to unlabeled data, transforming it into a two-class classification task. After building the model, we can determine the most informative features by calculating the variable importance.

Two methods commonly used for this purpose are permutation-based and Gini impurity-based variable importance. In the permutation-based approach, increases in prediction error are calculated by shuffling the out-of-bag data for a feature while leaving all other variables intact. Alternatively, the Gini impurity-based approach measures how effectively a feature purifies the nodes in the decision trees. In RFs, node splitting on variables ensures that offspring nodes are less impure. The variable importance is calculated by summing the reductions in Gini impurity across all trees. A larger reduction in impurity indicates greater importance [69]. Once variable importance has been calculated, the variables can be sorted to select those with the highest scores for subsequent computational or biological analysis. This approach could be applied for both computational and biological purposes.

#### Stability selection

Stability selection is a feature selection method that combines subsampling with variable selection to restrict the number of false discoveries in the set of selected variables [73]. It operates by repeatedly subsampling the data and the features and running a feature selection algorithm, such as Lasso or RF, on each subset. This process is repeated multiple times to collect selected features for each subset. Finally, it aggregates the selected features across all runs, typically using a threshold to determine stable features. Stability selection identifies features chosen consistently across different subsets without relying on labeled data and is likely to be more stable and informative. Stability selection works well in high-dimensional sparse data [74, 75], especially in scenarios where the number of features exceeds the number of samples, reducing the risk of overfitting or addressing computational constraints. Stability selection can also effectively filter out irrelevant variables in datasets that are noisy or contain redundant features. It can provide reliable results even with small sample sizes. From a computational standpoint, stability selection can efficiently handle large datasets with many features by subsampling the data and features [76]. On the other hand, it may need to be improved in scenarios with strong correlations between features, as it may fail to differentiate between them. Additionally, determining the appropriate threshold for selecting stable features can be subjective and requires manual tuning [77, 78]. Stability selection has been applied to create transcription factor-DNA binding specificity models [79] and analyze DNA methylation data to identify and characterize DNA methylation sites associated with gestational age prediction [80].

#### Application to single-cell data

Features selected by RF or stability selection are valuable in single-cell analysis. RF identifies genes that best distinguish cells, aiding in pinpointing key markers for cell types or states. Importance scores prioritize genes driving meaningful variation, reducing noise for better downstream analysis. Stability selection emphasizes consistently chosen genes across datasets, helping to mitigate overfitting and noise. Combined, these approaches produce reliable and interpretable gene sets that aid in resolving cell populations, mapping developmental trajectories, and uncovering biological variation within single-cell data.

### Correlation and information-theoretic methods

#### Correlation-based approaches

Many correlation-based feature selection methods are supervised, focusing on features that strongly correlate with a target variable while minimizing redundancy between the features [81]. However, unsupervised strategies, such as AutoSOME and other tools, have also been explored to extract informative gene subsets without relying on predefined labels [82]. Some unsupervised techniques involve conceptualizing each gene as a potential network hub. Such methods identify genes that exhibit the highest number of strong correlations with other genes within the dataset [83, 84]. These genes are assumed to carry broader information content, whereas genes with weak or few correlations are removed as redundant. This approach is expected to produce a concise but highly informative subset suitable for downstream analysis.

Alternatively, a network-based approach begins by constructing a correlation matrix of all genes, represented as a graph where nodes correspond to genes and edges reflect pairwise correlation strength. Network analysis techniques, such as weighted gene coexpression network analysis, are then applied to cluster genes and identify central, highly connected ‘hub’ genes based on metrics such as degree centrality [85]. These hub genes often represent key regulators or integrators of genetic activity and can enhance the biological interpretability of feature selection [86, 87]. Hub genes identified through correlation-based analysis are key features in single-cell data and serve as central regulators or markers. They reduce dimensionality while preserving biological signals; improve clustering, trajectory inference, and cell-type classification; and enhance interpretability by linking phenotypes to molecular mechanisms.

#### Maximal information coefficient

The maximum information coefficient (MIC) selects relevant features by evaluating the strength of the relationship between each pair of variables in the data without assuming any specific relationship or functional form. This allows it to capture complex relationships, including linear and nonlinear dependencies [88]. The MIC aims to find the maximum dependency between two variables by systematically exploring all the data partitions and selecting the partition that maximizes mutual information.

The algorithmic steps are as follows: First, the data are discretized to convert continuous variables into discrete categories. Then, the data are partitioned into a grid of different resolutions. The mutual information is calculated for each possible pair of variables at each resolution. The pair with the maximum mutual information is selected as the maximal information coefficient. The process is repeated for finer resolutions until the maximum value is obtained. The MIC values are subsequently normalized to allow for a fair comparison across grids of different dimensions and to produce modified values within the range of 0–1 [88]. These MICs can be used to identify relevant genes associated with a particular phenotype or biological process using genomic data.

The MIC algorithm scales well with the number of variables, making it suitable for high-dimensional biological data analysis. The algorithm not only provides a measure of the strength of the relationship, allowing for the ranking and selection of features, but also has the potential to identify novel relationships. This is due to its capacity to capture a broad spectrum of functional and nonfunctional associations [89]. The MIC can be used to analyze both numeric and categorical data. This approach will enable future applications of the method to diverse biomedical datasets [90].

While the MIC algorithm offers significant benefits, it is important to acknowledge its limitations. Determining appropriate parameters, such as grid resolution and the number of partitions, can be challenging and may require careful tuning. Additionally, the MIC may not perform well in scenarios where the relationships between variables are very weak or when confounding factors are present.

#### Application to single-cell data

Features selected via correlation-based methods or MIC are highly useful in single-cell analyses. Correlation approaches identify hub genes and key coexpressed modules, capturing central regulators and essential biological processes. At the same time, MIC uncovers both linear and nonlinear relationships, detecting subtle or complex gene associations. Ranking genes by network centrality or MIC scores produces concise, informative feature sets that retain meaningful biological signals. In single-cell analyses, features clarify clusters, reveal cell progressions, and highlight regulatory networks, linking molecular signatures to observable phenotypes for further study.

### Generative and deep learning-based methods

#### Generative adversarial network

A generative adversarial network (GAN) is a ML model in which two neural networks, the generator and discriminator, engage in competitive deep learning to increase prediction accuracy. Features revealing crucial aspects can be obtained from the discriminators or the generator’s intermediate layers. Features from the generator capture data distributions and patterns, which are useful for generating synthetic data, whereas features from the discriminator capture discriminative information that distinguishes actual from generated samples, providing insights into important discriminatory aspects of the data [91]. The GAN’s unsupervised model operates within a competitive zero-sum game framework, where gains for one network correspond to losses for the other. However, unlike static loss functions, the trainable discriminator dynamically distinguishes between generated and authentic solutions, and a training signal for the generative part can be derived. Generative models craft training data resembling real examples. The discriminator learns to distinguish between generated and real data. Swift detection of errors leads to penalties for the generator, encouraging better output quality. During the training process, the generator aims to minimize the error rate of the discriminator by producing increasingly realistic synthetic data. Simultaneously, the discriminator strives to maximize its ability to differentiate between real and artificial samples. This dynamic interplay drives both networks to continuously improve, ultimately enhancing the overall performance of the GAN [91].

Machine learning models based on GANs have several applications in transcriptomics and biology, such as improving imputation performance across both major and rare cell populations, offering a solution to enhance the recovery of biologically meaningful expression patterns in scRNA-seq datasets [92], altering semantically unique aspects of cellular identity and forecasting individual cellular gene expression responses to drug treatment (MichiGAN) [93], and even providing an integration algorithm for eliminating nonbiological differences between different batches (IMGG framework) [94]. Some modifications of this model, including a two-stage training process and the utilization of multiple GANs to achieve cell-specific imputation, are proposed to further increase the accuracy of imputation, cell clustering, differential gene expression analysis, and trajectory analysis. These methods are scalable to large scRNA-seq datasets and consistently perform well across sequencing platforms [95].

#### Variational autoencoders

The variational autoencoder (VAE) is a neural network-based generative model designed to encode and decode high-dimensional data within a continuous latent space [96, 97]. While encoders reduce data dimensionality, decoders reconstruct the original data. Unlike conventional autoencoders, VAEs generate two vectors, means and standard deviations, rather than a single vector of size n, initiating the decoding process by sampling from a typically Gaussian distribution [98]. The VAE identifies relevant features through the latent space by learning to reconstruct the original data. This regularization enables the model to learn a smooth representation of the data, which is beneficial for tasks such as generating new data samples and interpolating between points. This generative model has been applied to image processing, text data, and bioinformatics [99, 100].

VAEs are trained to prioritize the most important features and filter out less prominent features from the latent space by minimizing reconstruction loss, i.e. the mean squared error between the input and reconstructed data. VAEs may compromise between generating realistic data and generating realistic counterfactual data [96]. Additionally, VAEs are computationally intensive in training and sensitive to hyperparameters. Ongoing research shows promise in alleviating these challenges, such as refining the model architecture and optimizing to enhance computational efficiency [98] and reduce sensitivity to hyperparameters [101]. VAEs have been applied to learn latent molecular structure representations [102], facilitating drug discovery [103], and protein folding prediction tasks [104]. Furthermore, VAE models can be integrated with other computational methods and algorithms to perform clustering, dimensionality reduction, and trajectory inference to identify cell types, states, and trajectories via single-cell RNA sequencing data [105–107].

#### Application to single-cell data

Features extracted from generative and deep learning models, such as GANs and VAEs, are highly valuable for single-cell data. GANs extract features from discriminators or generators that highlight key patterns and discriminative signals, which are helpful for tasks such as imputing missing data, correcting batch effects, and enhancing clustering or trajectory analyses. VAEs capture the most informative features in their latent space, creating smooth, low-dimensional representations that preserve biological variation. By selecting genes or components based on latent embeddings or reconstruction importance, these approaches help pinpoint distinct cell states, active gene programs, and transitional processes, providing a foundation for reliable downstream analysis and enhanced interpretability of single-cell landscapes.

### Clustering-based methods

#### Hierarchical clustering

Hierarchical clustering is a popular method for grouping data points based on their similarity. This method creates a hierarchy of clusters, represented by a dendrogram, where each node represents a cluster [108]. There are two main approaches to hierarchical clustering: agglomerative and divisive. Agglomerative clustering starts with individual data points as separate clusters and progressively merges them until a single cluster remains. On the other hand, divisive clustering begins with all the data points in one cluster and recursively divides them into smaller clusters. Divisive clustering continues until each data point forms its own cluster or until the desired number of clusters is achieved. Various approaches, such as single linkage, average linkage, weighted linkage, centroid linkage, median linkage, and Ward’s method, can be used to calculate the distance between clusters [109].

Cabezas *et al.* proposed a new method that treats the dendrogram as a phylogeny to use the full structure of the dendrogram [108]. This allows for visualizing feature segmentation and scoring feature importance. The phylogenetic feature importance score evaluates how well a feature contributes to the segmentation of a dendrogram based on an evolutionary model. It measures the inaccuracy of feature-based predictions over the dendrogram’s leaves, with higher scores indicating features that better explain the clustering structure, similar to the coefficient of determination (R^2) in regression analysis. The proposed method is implemented in R as PhyloHclust. Another approach is to hierarchically cluster the features, identify feature clusters, rank the features within each cluster, such as by variance, and select the top features from each cluster. This approach is useful for exploring relationships among features.

Overall, hierarchical clustering is a powerful and flexible technique with diverse methods for calculating distances between clusters. One of its key advantages is its flexibility, as it does not require prespecifying the number of clusters, allowing users to cut the dendrogram at the desired level to obtain meaningful clusters. However, determining which hierarchical clustering method to use and the optimal number of clusters can be challenging [109].

#### K-means

K-means is a popular unsupervised clustering method that groups data based on feature similarity [105]. It initializes k centroids, assigns points to the nearest centroid, recalculates centroids, and iterates these steps until convergence, typically when the cluster assignments no longer change or when the intracluster variance (also known as the within-cluster sum of squares) is minimized [110].

K-means is primarily a clustering algorithm, but it can also be extended for feature selection. One such approach is kernel penalized K-means (KPKM), which integrates feature selection into the clustering process by identifying the most informative features while preserving the original cluster structure. The KPKM enhances the standard K-means by incorporating kernel functions, allowing for more flexible, nonlinear separation of data. This framework enables the algorithm to achieve high-quality clustering using only a subset of relevant features, improving both interpretability and performance [111]. Another example is where feature selection was performed by combining a trace ratio formulation with k-means; the resulting framework (named TRACK) was not only able to identify the relevant features of a dataset but also to do so in an unsupervised way. This algorithm is called Unified Trace Ratio Formulation and K-means Clustering (TRACK) [112].

In terms of selecting the best set of features for clusters, several strategies can adapt K-means clustering for unsupervised feature selection. One approach involves analyzing cluster centroids after K-means to identify features with high variance across centroids, indicating their importance in distinguishing between clusters. Another method clusters the features themselves, such as genes, across samples, allowing the identification of coregulated or redundant features. Additionally, a wrapper-style approach iteratively selects subsets of features, applies K-means clustering, and evaluates the clustering quality to retain features that contribute most to effective cluster separation.

#### Application to single-cell data

Features selected via clustering methods like hierarchical clustering and K-means are valuable for single-cell data analysis. Hierarchical clustering identifies genes driving dendrogram structure and cellular hierarchies, while K-means highlights genes that distinguish clusters by capturing high-variance patterns. Selected features streamline single-cell analyses by clarifying cell-type structure, revealing developmental paths, and uncovering regulatory mechanisms, while simplifying high-dimensional data for more intuitive interpretation.

## Applications in computational steps

Conventional single-cell RNA-seq pipelines often begin by selecting HVGs, those exhibiting the largest per-gene dispersion across cells, as inputs for PCA and clustering [113]. While HVG selection is computationally efficient, it remains gene-centric, treating each gene independently and capturing only linear variance. This approach could enrich nonspecific transcripts while overlooking low-variance but biologically critical regulators. Moreover, technical noise can inflate variance, leading to HVG panels that propagate artifacts and could result in unstable cluster assignments and overcrowded, noisy two-dimensional embeddings.

Machine learning-based unsupervised feature selection addresses these limitations by evaluating genes in a multivariate context, capturing complex, nonlinear relationships. For example, methods that extract latent variables rank genes based on their contributions to latent expression programs, retaining features that reconstruct the whole expression space while filtering out redundant or noisy signals [114]. Similarly, mutual information-based approaches and stability-selection methods could prioritize genes that maximize the preservation of manifold structure, enhancing silhouette scores and improving the resolution of subtle cell states. Deep learning models and diffusion map-based graphs learn nonlinearly. Using these scores as feature sets could produce clearer UMAP visualizations, sharper pseudotemporal trajectory branches, and fewer spurious clusters [44, 115]. These methods excel at denoising high-dimensional data, improving the signal-to-noise ratio, and enabling the detection of rare cell populations or transient states that HVGs may miss.

In addition to improving the accuracy of downstream analyses, unsupervised feature selection significantly reduces computational overhead. Pruning the initial gene set from ~20 000 to a few hundred informative features lowers the memory requirements and accelerates the construction of nearest-neighbor graphs and clustering algorithms. This efficiency extends to integrative tools, such as Harmony or weighted nearest-neighbor methods for multiomic mapping, which benefit from compact, denoised input matrices [116].

Unsupervised feature selection also enhances downstream modeling by providing robust initializations and reducing the likelihood of overfitting. In supervised tasks, such as cell-type classification, feature sets derived from unsupervised methods reduce model complexity, mitigate overfitting, and improve generalization to unseen data. Additionally, these methods can uncover biologically meaningful gene modules, such as coregulated genes or regulatory networks, which are more interpretable than HVG-derived features and align with underlying biological processes [114].

In summary, ML-driven unsupervised feature selection delivers cleaner input matrices, more faithful low-dimensional representations, and faster, more stable downstream computations. By incorporating these modern selectors early in the scRNA-seq workflow, researchers can enhance computational efficiency, enabling more robust analyses of cellular heterogeneity and dynamic processes.

## Applications in biology

Supervised learning methods are widely used in single-cell analysis, with DEG analysis being popular for comparing clusters or experimental groups, such as disease versus healthy individuals. These methods can cause information loss if clustering is too coarse or fails to account for intracluster heterogeneity, simplifying continuous processes into binary comparisons [117]. For example, when one cluster is compared with the remaining clusters, the group of remaining clusters consists of diverse cells, and the patterns among them are easily masked. Unsupervised techniques can reveal complex, overlapping, or gradual expression patterns across clusters, detecting subtle variations and dynamic transitions that might be missed by traditional DEG testing. After identifying patterns, they can be linked to cell types and metadata, but any classification does not limit pattern recognition. DEG analysis is also gene-centric and may miss broader coexpression patterns or latent programs that drive cellular behavior. These limitations can further hinder discovery and introduce bias when labels are incomplete or incorrect.

Supervised methods rely heavily on predefined labels, which may not fully capture biological complexity, especially in systems with continuous transitions, overlapping cell states, or rare populations [118, 119]. In contrast, unsupervised ML offers a rich framework for uncovering patterns in high-dimensional single-cell data without prior annotations. These approaches not only provide interpretable, generalizable features across datasets but also support hypothesis generation, complementing the hypothesis-testing nature of supervised tools. Together, these methods provide a more nuanced and data-driven understanding of cellular heterogeneity.

To synthesize the strengths, limitations, and feature selection strategies of the discussed unsupervised methods, we provide a comparative summary in Supplementary Table S1. This overview highlights how each method contributes uniquely to single-cell data analysis, setting the stage for the following case study, which illustrates the practical application of one such approach.

## Case study

To illustrate the practical application of unsupervised feature selection methods, we selected one representative technique and applied it to a previously analyzed single-cell RNA-seq dataset. The aim of this analysis was not to conduct an exhaustive evaluation but rather to provide a concise example demonstrating how such methods can uncover biologically meaningful features without relying on prior labels.

**Figure 2:**
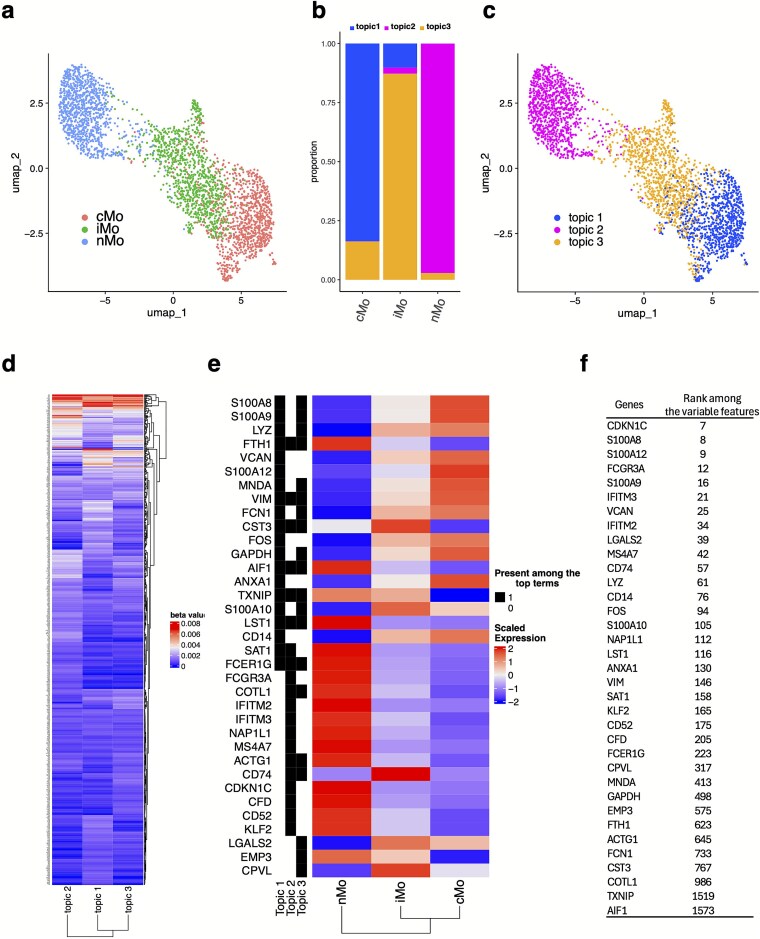
Representative example showcasing unsupervised feature selection in a single-cell transcriptomic dataset. (a) UMAP plot showing three major monocyte populations: classical (cMo), intermediate (iMo), and non-classical (nMo), used for topic modeling in this analysis. (b) Distribution of topic proportions across monocyte subsets, showing how each cell type is characterized by a mixture of the three identified topics. (c) UMAP plot visualization with cells colored by their dominant topic assignment, illustrating how learned topics align with biological structure in the data. (d) Beta values (topic-specific feature weights) for genes across the three topics, representing the strength of each gene’s contribution to individual topics. (e) Top 20 genes with the highest beta values from each topic, providing insight into topic-specific gene signatures. Because some genes appeared across multiple topics, the final list contained 35 unique genes. The binary heatmap on the left indicates the presence of each gene within each topic: black denotes presence, white denotes absence. (f) Rankings of the top topic-model-selected genes by their expression variability, illustrating differences between unsupervised topic modeling and traditional variable gene selection approaches.

For this purpose, we selected topic modeling, specifically LDA. We applied it to a single-cell RNA-seq dataset previously published [120] and reanalyzed [121] to study monocyte differentiation and diversity. This dataset comprises three monocyte subsets from healthy individuals: classical monocytes (cMo), intermediate monocytes (iMo), and nonclassical monocytes (nMo) (Fig. 2a). Detailed information regarding sample collection, processing, and initial analyses can be found in the original publications [120, 121].

We performed this analysis using the topicmodels [53] package in R. Normalized gene expression data were used as input, and the LDA model was fitted with standard parameters as described in the package documentation. We selected three topics, aiming to capture latent structures corresponding to the three known monocyte subsets (Fig. 2b). The cells were assigned to their dominant topic based on the highest topic proportion. This simple matching approach revealed a strong correspondence between the inferred topics and known cell types (Fig. 2b and c), despite the fully unsupervised nature of the method. Minor discrepancies between topics and annotated cell types may reflect biological heterogeneity or smaller subpopulations not captured by limiting the model to three topics. Nevertheless, the method effectively distinguished the major monocyte classes, which are known to share extensive transcriptional overlap.

We next extracted the top 20 genes with the highest beta coefficients (topic-specific feature weights) from each topic. This overlap resulted in 35 unique genes across all the topics (Fig. 2d and e). Visualization of their expression across the three cell types revealed clear topic-specific patterns for many of these genes (Fig. 2e), several of which have previously been reported as markers of monocyte subsets [121, 122]. This finding is particularly notable, as the topic modeling approach was performed without access to any cell type annotations but was still able to recover biologically relevant features.

Finally, we assessed how these topic-selected genes ranked among the highly variable genes identified via traditional variance-based methods (Fig. 2f). Interestingly, approximately two-thirds of the genes identified by the LDA model were not among the top 100 variable genes, with some falling outside the top 500. This suggests that conventional approaches that rely solely on statistical dispersion may overlook biologically informative genes, especially in high-dimensional, noisy data. If downstream analyses are restricted to a small subset of top variable genes, functionally relevant markers may be inadvertently excluded. This underscores the value of unsupervised ML methods as complementary or even superior alternatives for feature selection in single-cell analyses.

It is important to note that while most workflows retain the top 1000–2000 highly variable genes out of ~20 000, biologically critical genes with moderate variance, such as transcription factors or regulatory molecules, may fall outside this range and be excluded from analysis. For instance, a gene essential for monocyte transition might not appear among the top variable genes but can still be identified through unsupervised methods such as topic modeling. This demonstrates the strength of such approaches in recovering informative, low-variance features that may be overlooked by traditional variance-based filtering, particularly in transitional populations like intermediate monocytes.

## Future directions

Future advancements in unsupervised feature selection for single-cell data will likely emphasize the integration of multiomic information to uncover complex biological patterns. While this review emphasizes feature selection methods applied to individual omic modalities, integration of multiomic datasets represents a major challenge in single-cell analysis. The algorithms discussed can be applied independently to each modality to identify informative features, which can then be used for downstream multimodal analyses. Developing methods that perform feature selection directly across multiple modalities requires specialized approaches to handle differing data distributions and scales. Addressing this problem is an active area of research, and its complexity suggests that a comprehensive review of multimodal feature selection could form the basis of a separate, dedicated study.

Scalable algorithms are crucial for handling large datasets, striking a balance between computational efficiency and interpretability. The incorporation of domain knowledge, such as gene regulatory networks or pathways, may guide feature selection, thereby boosting biological relevance. Moreover, standardizing benchmarking frameworks and developing open-source toolkits will be crucial for evaluating method performance across diverse datasets, promoting reproducibility and widespread adoption in single-cell research. Finally, foundation models trained on vast biomedical corpora could act as priors that inform or refine unsupervised selectors, helping to highlight genes whose functions or interactions are underrepresented in the data yet well documented in the literature.

## Conclusion

Unsupervised feature selection empowers data-driven discovery in single-cell data, revealing patterns without predefined labels. Techniques such as dimensionality reduction, latent factor models, and ensemble methods extract biologically relevant features, thereby enhancing clustering, visualization, and trajectory inference. By reducing dimensionality, these approaches improve efficiency and uncover hidden structures. However, challenges such as sparsity, noise, and scalability persist, necessitating future innovations in multiomic integration and domain-informed algorithms. As single-cell technologies advance, unsupervised feature selection will be pivotal in decoding cellular heterogeneity, offering new insights into health and disease, and transforming biological research through unbiased, interpretable analyses.

**Key Points**

- Single-cell technologies generate high-dimensional, noisy datasets where conventional feature selection (e.g. highly variable genes) can bias results and overlook critical biological signals.
- Model-based feature selection via unsupervised machine learning methods enables unbiased discovery of informative features by capturing complex patterns without relying on predefined labels
- These approaches significantly enhance downstream analyses, such as clustering and visualization, by improving the signal-to-noise ratio and revealing biologically meaningful insights, while improving computational efficiency.
- Applications in biology demonstrate that unsupervised feature selection can uncover subtle cell states, rare populations, and biologically relevant gene modules often missed by traditional strategies.
- Future directions emphasize integrating multiomic data, developing scalable and interpretable algorithms, and incorporating domain knowledge to advance single-cell data analysis.

## Supplementary Material

Supplementary_Table_1_bbag006

## Data Availability

The scRNA-seq dataset used in this study was downloaded from the NCBI Gene Expression Omnibus (GEO) database and is publicly available under series accession number GSE214546.
